# Laboulbeniales (Fungi: Ascomycota) infection of bat flies (Diptera: Nycteribiidae) from *Miniopterus schreibersii* across Europe

**DOI:** 10.1186/s13071-018-2921-6

**Published:** 2018-07-05

**Authors:** Tamara Szentiványi, Danny Haelewaters, Walter P. Pfliegler, Laura Clément, Philippe Christe, Olivier Glaizot

**Affiliations:** 1 Department of Ecology and Evolution, University of Lausanne, Biophore, CH-1015 Lausanne, Switzerland; 2 Museum of Zoology, Palais de Rumine, Place de la Riponne 6, CH-1014 Lausanne, Switzerland; 3 Department of Organismic and Evolutionary Biology, Harvard University, Cambridge, Massachusetts 02138 USA; 4 Farlow Reference Library and Herbarium of Cryptogamic Botany, Harvard University, 22 Divinity Avenue, Cambridge, Massachusetts 02138 USA; 5 Department of Biotechnology and Microbiology, University of Debrecen, Egyetem tér 1, Debrecen, H4032 Hungary

**Keywords:** *Arthrorhynchus*, Bat flies, Ectoparasites, Host specificity, Laboulbeniales, Nycteribiidae

## Abstract

**Background:**

Bat flies (Diptera: Nycteribiidae and Streblidae) are obligate, blood-sucking ectoparasites of bats with specialized morphology, life-cycle and ecology. Bat flies are occasionally infected by different species of Laboulbeniales (Fungi: Ascomycota), microscopic fungal ectoparasites belonging to three genera: *Arthrorynchus* spp. are restricted to the Eastern Hemisphere, while species of *Gloeandromyces* and *Nycteromyces* occur on Neotropical bat flies. Little is known about the distribution and host specificity of *Arthrorynchus* spp. on bat flies. In this study, we focused on sampling bat flies from the cave-dwelling bat species *Miniopterus schreibersii*. Bat and ectoparasite collection took place in Albania, Croatia, Hungary, Italy, Portugal, Slovakia, Spain and Switzerland. Flies were inspected for Laboulbeniales infections.

**Results:**

Six hundred sixty seven bat flies of five species were collected: *Nycteribia latreillii*, *N. pedicularia*, *N. schmidlii*, *Penicillidia conspicua*, and *P. dufourii*. Laboulbeniales infection was observed on 60 specimens (prevalence = 9%). Two Laboulbeniales species, *Arthrorhynchus eucampsipodae* and *A. nycteribiae*, were present on three bat fly species. All observations of *A. eucampsipodae* were on *N. schmidlii*, and *A. nycteribiae* was present on *P. conspicua* and *P dufourii*. *Arthrorhynchus eucampsipodae* is, for the first time, reported from Slovakia and Spain. *Arthrorhynchus nycteribiae* represents a new country record for Portugal and Slovakia. There were no significant differences among infection rates in different countries. Females of *N. schmidlii* showed a higher infection rate than males with an observable trend (*P* = 0.0502). No sex differences in infection rate for *P. conspicua* and *P. dufourii* were detected. Finally, thallus density was significantly lower in *N. schmidlii* compared to *P. conspicua* and *P. dufourii*.

**Conclusions:**

With this study, we contribute to the knowledge of the geographical distribution and host specificity of Laboulbeniales fungi associated with ectoparasitic bat flies within Europe. We discuss parasite prevalence and host specificity in the light of our findings and the available literature. *Penicillidia conspicua* is unambiguously the main host species for *A. nycteribiae* based on our and previous findings. Differences in parasite intensity and sex-biased infections of the fungi are possible depending on the species.

**Electronic supplementary material:**

The online version of this article (10.1186/s13071-018-2921-6) contains supplementary material, which is available to authorized users.

## Background

The distribution of parasites is shaped by the distribution of their hosts, although complete overlaps are infrequent. Hosts can lose their parasites or gain new ones when colonizing new areas [1, 2]. Whether parasites are lost or gained is driven by a combination of abiotic and biotic factors. Abiotic factors, such as climate or habitat type can strongly affect parasite occurrence [3]. Biotic factors, for instance host behavior or immune response to parasitism, may be essential in determining factors in parasite distribution [4, 5]. Studying geographical differences in parasite distributions is the first step in understanding how parasite loss or gain is shaped.

Bats represent the second largest mammal order with a worldwide distribution and have highly specific and diverse micro- and macroparasites [6], which can be subject to parasites of their own [7]. These multilevel trophic systems may be shaped by many parameters, such as the ecology, immunology, behavior and sex of the bat hosts as well as their parasites (e.g. [4, 8, 9]).

This study focuses on *Miniopterus schreibersii*, a cave-dwelling bat which is widely distributed across southern Europe, Asia Minor and North Africa, and represents the only European member of its genus [10]. *Miniopterus schreibersii* hosts a myriad of highly specific parasites as for example *Spinturnix psi* mites (Acari: Mesostigmata: Spinturnicidae), *Nycteribia schmidlii* and *Penicillidia conspicua* bat flies (Diptera: Hippoboscoidea: Nycteribiidae), or *Polychromophilus melanipherus* blood parasites (Alveolata: Apicomplexa: Plasmodiidae) [9, 11, 12]. Additionally, non-specific ectoparasites, such as the bat fly species *Penicillidia dufourii*, *Nycteribia latreillii* and *N. pedicularia*, can parasitize *M. schreibersii*. Even though these non-specific associations are considered the result of accidental host choice by the parasites, they cannot be considered rare [9]. Altogether, *M. schreibersii* represents an outstanding target species in parasitology research.

Bat flies are obligate blood-sucking ectoparasites of bats belonging to two families, the Nycteribiidae and the Streblidae. In Europe, 16 nycteribiid and one streblid species have been reported so far [9]. The morphology and life-cycle of bat flies are unique among Diptera. Nycteribiids are wingless and possess reduced ocelli, both being adaptations for living in the fur of their hosts. Bat flies give birth to a single third-instar larva (larviposition) on the roost wall of their hosts. Before larviposition, the larva develops in the uterus-like organ of the female, nourished by milk-glands. The larva immediately pupates after larviposition and the emergence of imagoes from pupae is influenced by the presence of bats; after emergence bat flies actively search for bat hosts [6].

Laboulbeniales are ectoparasitic fungi that associate with representatives of three subphyla of Arthropoda: Chelicerata, Myriapoda and Hexapoda [13]. Of all described Laboulbeniales species, 80% are associated with Coleoptera and 10% with Diptera. The rest occur on many other different taxa, belonging to Arachnida, Diplopoda and Hexapoda [13]. The Laboulbeniales are different from most other fungal groups because they lack hyphae and instead form microscopic fruiting bodies or *thalli* as a result of determinate growth. Most Laboulbeniales are moderately to extremely host-specific. Many members of the order are associated with a single host species or several species of a same genus. Some species exhibit position specificity and are found on determined parts of their host’s integument [14]. What drives host specificity in nature is unknown. Ecological specificity is the last but most interesting level; shifts between phylogenetically unrelated hosts that share the same microhabitat is a significant trigger for speciation [15–17]. Three genera and eight species of Laboulbeniales are known from bat flies [7, 18], with the potential of many undescribed species, especially in the Neotropics [19]. The genera *Gloeandromyces* and *Nycteromyces* are reported on streblid bat flies from Central and South America. *Arthrorhynchus* is apparently restricted to the Eastern Hemisphere and has only been reported from Nycteribiidae. Four species are known: *Arthrorhynchus acrandros*, *A. cyclopodiae*, *A. eucampsipodae* and *A. nycteribiae* (although *A. acrandros* is disputed [7]). *Arthrorhynchus nycteribiae* has been most widely reported [7, 19]. It is known in Europe (Austria, Bulgaria/Slovakia, Croatia, the Czech Republic, “Czecho-slovakia” [sic.], Denmark, France, Hungary, Italy, Poland, Romania, Russia, Serbia, Spain, Sweden, Switzerland and the Netherlands), Africa (Kenya and Zambia), Asia (Sri Lanka) and Oceania (Australia).

In a recent study, 1494 bat flies (11 spp.) from 1594 bats (28 spp.) collected in Europe were screened for the presence of Laboulbeniales fungi [7]. Many bat flies parasitized by these fungi have been collected from *M. schreibersii*. The prevalence of Laboulbeniales on bat flies from this species was highly disparate among bat fly species. *Nycteribia schmidlii* had a parasite prevalence of 3.1% (*n* = 147), and was infected by both *Arthrorhynchus eucampsipodae* and *A. nycteribiae*. *Penicillidia conspicua* had a prevalence of 23.1% (*n* = 142); only *A. nycteribiae* was found. All *P. dufourii* from *M. schreibersii* were found to be uninfected (*n* = 22).

In the present study, we expanded capturing efforts of *M. schreibersii* to focus on Laboulbeniales infections of specific *versus* generalist bat flies. Using this tripartite system, we attempted to assess parasite distributions within and between host populations.

## Methods

### Collection of bats and bat flies

**Fig. 1:**
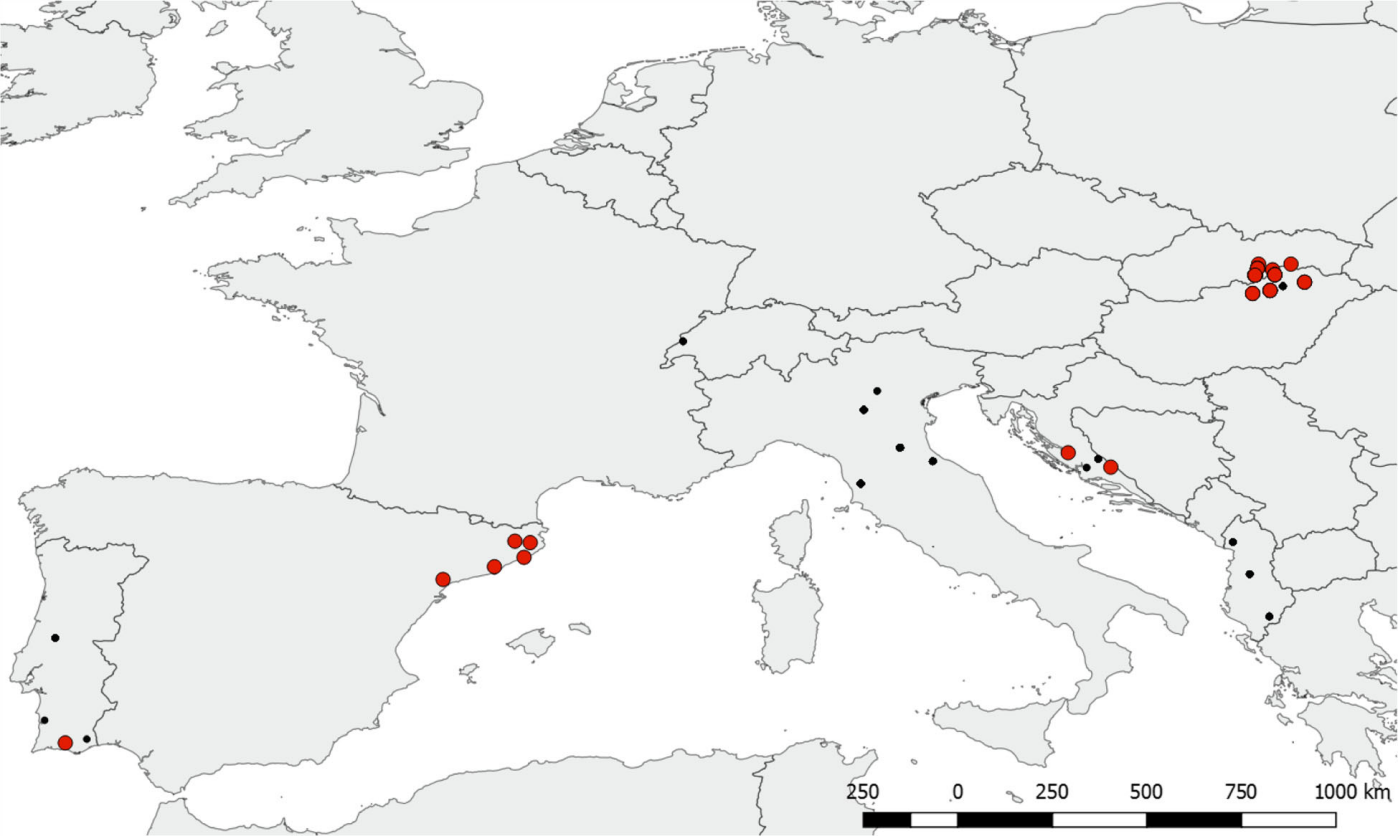
Map of the collection sites. Laboulbeniales infected populations are indicated with red dots, non-infected bat fly populations are indicated with black. Infection was found in Croatia, Hungary, Portugal, Slovakia and Spain

Bats were captured from April through September in 2009–2016, using mist nets and harp-traps, placed in front of caves where *M. schreibersii* colonies occur. Sampling took place in Albania, Croatia, Hungary, Italy, Portugal, Slovakia, Spain and Switzerland (Fig. 1). Exact localities are given in Additional file 1: Table S1. Age, sex, and morphological characteristics were collected for most individuals. Bat flies were removed with forceps and placed in 70–90% ethanol. Bats were released immediately after processing in the vicinity of the capture site.

Bat fly species and sex were determined following Theodor’s key (1967) [21]. Voucher bat fly specimens are deposited under the accession number 16CH12-XB07 at the Museum of Zoology, Lausanne, Switzerland.

### Collection and identification of Laboulbeniales

The presence or absence of Laboulbeniales was determined using a stereomicroscope (Leica M205C, Leica Mcrosystems AG, Heerbrugg, Switzerland). For each infected bat fly, the total number of Laboulbeniales thalli was counted. Thalli were removed from the host at the point of attachment with an entomological pin and slide-mounted in Amman’s solution for identification [22]. Identification was based on the original descriptions and drawings by Thaxter [23] and recent amendments by Haelewaters et al. [7]. Slides will be deposited at the mycology herbarium of Ghent University, Belgium (Ghent).

### Statistical analyses

Fisher’s exact tests were used for prevalence comparison among countries, bat fly species and sexes. In addition, Mood’s median tests were performed to compare the median thallus density and bootstrap tests were used to compare mean thallus density of Laboulbeniales among infected bat fly hosts (based on 1000 bootstrap replications), performed in Quantitative Parasitology v.3.0 [24].

## Results

### Bat flies and Laboulbeniales

We collected 667 bat flies from 270 *M. schreibersii* bats. Five bat fly species were encountered: *N. schmidlii* (*n* = 468), *P. conspicua* (*n* = 144), *P. dufourii* (*n* = 52), *N. pedicularia* (*n* = 2) and *Nycteribia latreillii* (*n* = 1). Of all bat flies, 60 specimens were infected with Laboulbeniales fungi (prevalence of 9%). *Nycteribia latreillii* and *N. pedicularia* were represented in very low sample numbers and were uninfected with Laboulbeniales, therefore we excluded them from further analyses, figures and tables. *Arthrorhynchus eucampsipodae* was found in Hungary, Slovakia and Spain (Table 1). All observations of *A. eucampsipodae* were made on a single bat fly host, *Nycteribia schmidlii*. Additionally, we reported *A. nycteribiae* from five countries: Croatia, Hungary, Portugal, Slovakia and Spain (Table 1). Hosts for *A. nycteribiae* were *P. conspicua* and *P. dufourii*. The presence of *A. eucampsipodae* is reported here, for the first time, from Slovakia and Spain. Additionally the presence of *A. nycteribiae* is also a new record from Portugal and the first undoubtful record from Slovakia.

**Table 1:** Host associations of *Arthrorhynchus eucampsipodae* and *A. nycteribiae* reported during this study

Country	No. of collected flies (overall prevalence in %)	No. of infected flies	No. of *Arthrorhynchus eucampsipodae*	Host	No. of *Arthrorhynchus nycteribiae*	Host
Albania	32 (0)	0	–		–	
Croatia	17 (11.7)	2	–		2	PCON
Hungary	82 (15.8)	13	3	NSCH	10	PCON, PDUF
Italy	327 (0)	0	–	–	–	
Portugal	11 (9)	1	–		1^a^	PCON
Slovakia	152 (25.6)	39	19^a^	NSCH	20^b^	PCON, PDUF
Spain	42 (11.9)	5	1^a^	NSCH	4	PCON
Switzerland	4 (0)	0	–		–	
Total	667 (8.9)	60	23		37	

*Abbreviations*: *NSCH* *Nycteribia schmidlii*, *PCON* *Penicillidia conspicua*; *PDUF* *Penicillidia dufourii*

^a^First country record

^b^First undoubtful country record

### Prevalence rate in different countries

In Albania, Italy and Switzerland, Laboulbeniales infection was not detected among the 361 collected specimens of *Nycteribia schmidlii*, *Penicillidia conspicua* and *P. dufourii*. The highest parasite prevalence was observed in Slovakia (25.6%). We found the lowest overall parasite prevalence in Portugal (10%, only *P. conspicua* sampled). In Croatia, Hungary and Spain, overall fungal prevalence was 11.7%, 15.8% and 11.9%, respectively. There were no significant differences in parasite prevalence between the different countries.

### Parasite prevalence and host specificity

Of the 468 collected *N. schmidlii*, 23 flies were infected (4.9%) with Laboulbeniales. We sampled 52 specimens of *P. dufourii* of which 4 individuals carried Laboulbeniales (7.7%). Of 144 *P. conspicua* specimens, 33 were infected (22.9%). *Penicillidia conspicua* had a significantly higher parasite prevalence compared to the other two species (Fisher’s exact test, *P* < 0.0001). Infection by *A. eucampsipodae* was found exclusively on *N. schmidlii*, while *A. nycteribiae* infection was detected only on *Penicillidia* species.

### Differences in parasite prevalence between female and male bat flies

**Fig. 2:**
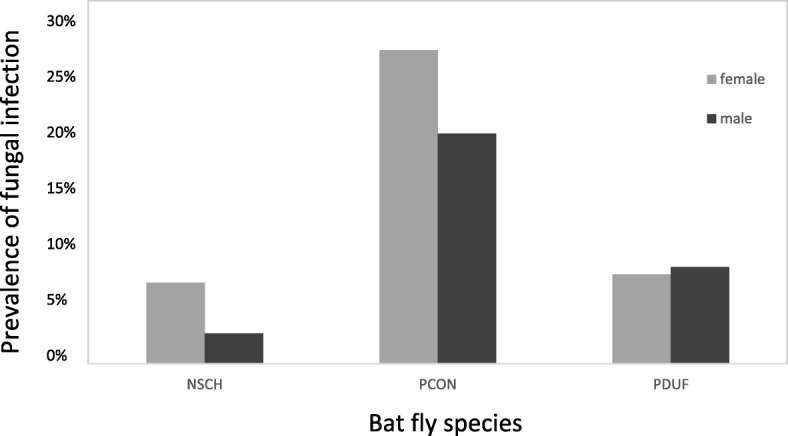
The infection rates of male and female bat flies across three species, *Nycteribia schmidlii* (*n* = 468; 269 females and 199 males), *Penicillidia conspicua* (*n* = 144; 81 females and 63 males), and *P. dufourii* (*n* = 52; 25 females and 27 males). Female flies are represented by gray, males with black bars. Prevalence rate of sexes is given (in percentage) for each category. *Abbreviations*: NSCH, *Nycteribia schmidlii*; PCON, *Penicillidia conspicua*; PDUF, *Penicillidia dufourii*

Of 269 females and 199 males of *N. schmidlii*, we found 18 infected females and 5 infected males, which shows a marginally significant trend in the infection between the sexes (6.7 and 2.5%, respectively; Fisher’s exact test, *P* = 0.0502). Of 81 females and 63 males of *P. conspicua*, 21 females and 12 males were infected (25.9 and 19%, respectively; Fisher’s exact test, *P* = 0.424) with Laboulbeniales. Of *P. dufourii*, 25 females and 27 males were collected and only two individuals were infected for each sex (8 and 7.4%, respectively; Fisher’s exact test, *P* = 1.0; Fig. 2), therefore the prevalence is not significantly different between the sexes, neither in *P. conspicua*, nor in *P. dufourii*.

### Thallus density of Laboulbeniales on bat flies

**Fig. 3:**
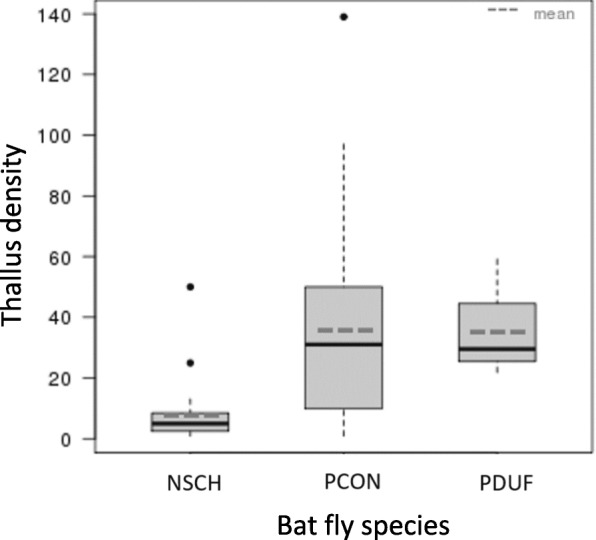
Thallus density of Laboulbeniales on *Nycteribia schmidlii* (*n* = 23), *Penicillidia conspicua* (*n* = 33) and *P. dufourii* (*n* = 4). Significant difference is observed between *N. schmidlii *and *Penicillidia* spp. (Mood’s median test, P < 0.0001)

The mean thallus density (± SD) per specimen was 7.8 ± 10.6 for *N. schmidlii* (*n* = 23), 35.6 ± 31 for *P. conspicua* (*n* = 33) and 35 ± 16.3 for *P. dufourii* (*n* = 4). *Nycteribia schmidlii*, which was infected with *A. eucampsipodae*, showed significantly lower thallus density when median intensities were compared among the three bat fly species (Fig. 3, Table 2; Mood’s median test, *P* < 0.0001). The thallus density was not significantly different between female and male bat flies (Table 2), neither for *N. schmidlii* (Bootstrap two-sample t-test, *P* = 0.223, female: 8.8 ± 11.7, male: 4.2 ± 3.5) nor for *P. conspicua* (Bootstrap 2-sample t-test, *P* = 0.499, female: 38.1 ± 35.2, male: 31.4 ± 22.4). *Penicillidia dufourii* was excluded from this analysis due to low sample size. *Nycteribia schmidlii* females (*n* = 18) showed a mean thallus density of 8.8, while males (*n* = 5) had a mean thallus density of 4.2. Mean thallus density on *P. conspicua* was 38.1 for females (*n* = 21) and 31.4 for males (*n* = 12) (Table 2).

**Table 2:** Mean and median thallus density of *Nycteribia schmidlii* and *Penicillidia conspicua* within species and between sexes

Species	Female		Male		Total			
	Mean density (*n*)	SD	Mean density (*n*)	SD	*n*	Mean density	Median density	SD
NSCH	8.8 (18)	11.7	4.2 (5)	3.5	23	7.87	5.0	10.6
PCON	38.1 (21)	35.2	31.4 (12)	22.4	33	35.6	31	31
PDUF	–	–	–	–	4	35	27.5	16.3

*Abbreviations*: *NSCH Nycteribia schmidlii*, *PCON Penicillidia conspicua*, *SD* standard deviation

## Discussion

### Geographical distribution, host range, and prevalence of Laboulbeniales

The geographical distribution and host range of bat-fly associated Laboulbeniales in Europe has been reported by several studies [7, 20, 25, 26]. Our study presents additional occurrence data focusing on the tripartite system of the *M. schreibersii* cave-dwelling bat, its ectoparasitic bat flies and their Laboulbeniales ectoparasites. Our findings agree with previously reported host species for these fungi [7, 19]. To date, *P. conspicua* has only been reported as host for *A. nycteribiae*, while *P. dufourii* and *N. schmidlii* are host for both *A. eucampsipodae* and *A. nycteribiae*.

Blackwell [20] reported the prevalence of *Arthrorhynchus* species to be 2.2% (*n* = 2517). Regarding the bat fly species discussed in our study, she found parasite prevalences of 2.5% on *N. schmidlii* (*n* = 316), 18.6% on *P. conspicua* (*n* = 86) and 0.7% on *P. dufourii* (*n* = 289). Haelewaters et al. [7] reported a total prevalence of 3% on all screened bat flies (*n* = 1494) but the parasite prevalence varied depending on the host species. These and our results allow us to suggest that these low (*N. schmidlii*, *P. dufourii*) to moderate (*P. conspicua*) infection rates in these species are not particularly variable.

Based on our study and the recent work by Haelewaters et al. [7], *A. eucampsipodae* seems to be highly specific towards *N. schmidlii*. However, Blackwell [20] reported other host species for *A. eucampsipodae* with prevalences ranging between 0.3–8.3%. Although *A. eucampsipodae* displays preferences for *N. schmidlii*, strict host specificity does not seem to be the rule for this Laboulbeniales species. The same specificity pattern is observed for *A. nycteribiae* [7, 19].

Based on our results, we can conclude that *Penicillidia dufourii* is merely a secondary host species for *A. nycteribiae* compared to *P. conspicua*. This confirms findings by Haelewaters et al. [7] who reported a prevalence for *A. nycteribiae* of 25% on *P. conspicua* (*n* = 152), whereas on *P. dufourii* prevalence was much lower (2.0%, *n* = 102). In addition, Blackwell’s [20] data, also show that *P. conspicua* has the highest prevalence with *A. nycteribiae* (18.6%, *n* = 86). Taken together, *P. conspicua* is unambiguously the main host species for *A. nycteribiae*, but this fungus probably has the capacity to also grow on many other bat fly hosts.

Although Laboulbeniales prevalence on bat flies varied among countries, we did not find significant differences. Since infection rates can be influenced by habitat type on a smaller geographical level, such as differences between wet or dry habitats (see [26] and references therein), future work should focus on identifying factors that shape the distribution of infection.

### Prevalence of Laboulbeniales between bat fly sexes

In parasitological studies, it is widely observed that different sexes often show different infection rates throughout several taxa [8, 27, 28]. Recently, it was found that among bat flies females are more likely to be infected with *Arthrorhynchus* spp. compared to males [7]. In our study, we only found a trend supporting this observation in the case of *N. schmidlii* (*P* = 0.0502), but no support was found for *P. conspicua* and *P dufourii* (Fig. 2). A commonly reported species of Laboulbeniales for which infection patterns can be significantly different between male and female hosts is *Hesperomyces virescens*, a parasite of ladybirds (Coleoptera: Coccinellidae). Sexual differences (e.g. prevalence and/or position specificity) in *H. virescens* infection on *Harmonia axyridis* ladybirds are presumed to be the result of host mating behavior [29–31]. Regarding bat flies, females are known to live longer [32]. Pregnant bat flies are significantly larger than male flies, accounting for more integument surface, and have fat reserves organized as lobes in their haemolymph, presenting higher nutritional resources [33].

Different sexes can exhibit different levels of parasite resistance [27, 33, 34]. These differences are most commonly explained by variances in hormone levels between sexes, for example steroid reproductive hormones [28]. In conclusion, sex bias in parasitism can occur also as a consequence of the different immune status of the hosts.

### Thallus density of Laboulbeniales on bat flies

Thallus density of Laboulbeniales can vary over time and can be different between host sexes and among host body parts [26, 35–37]. We found significantly higher thallus density on *P. conspicua* and *P. dufourii* compared to *N. schmidlii* (*P* < 0.001). This difference could be linked to host size, since both species of *Penicillidia* can reach body lengths of 3.4–4 mm, while *N. schmidlii* is much smaller (2–2.25 mm; [21]). However, *Arthrorhynchus* spp. also vary in size. *Arthrorhynchus eucampsipodae* (which parasitizes *N. schmidlii*) measures 375–550 μm in length (receptacle + cell VI + perithecium), while *A. nycteribiae* is generally longer, 390–750 μm [23, 38].

## Conclusions

During this study, we collected and analyzed the occurrence of *Arthrorhynchus* spp. in a wide range of geographical distributions within Europe on the cave-dwelling bat species, *Miniopterus schreibersii*. Five bat fly species were collected in eight countries, on which three species showed fungal infections, each with a different parasite prevalence. Prevalence can differ among host species and between host sexes. *Arthrorhynchus eucampsipodae* was only reported on *N. schmidlii*. In addition, *A. nycteribiae* was observed on two *Penicillidia* species, of which *P. conspicua* appeared to be the “main host” for this fungus, while *P. dufourii* is considered a secondary or accidental host. Our work has also resulted in new country records: *A. eucampsipodae* is newly reported from Slovakia and Spain, while *A. nycteribiae* is newly reported from Portugal and represents the first undoubtful record for Slovakia.

## Additional files

Additional file 1: **Table S1.** Additional data on bat, bat fly and Laboulbeniales occurrence. The exact location of parasite collection as well as sex and infection data of bat flies and fungus are shown. (XLSX 60 kb)
